# Test-retest reliability and agreement of lower-extremity kinematics captured in squatting and jumping preschool children using markerless motion capture technology

**DOI:** 10.3389/fdgth.2022.1027647

**Published:** 2022-12-05

**Authors:** Steen Harsted, Anders Holsgaard-Larsen, Lise Hestbæk, Ditte Lundsgaard Andreasen, Henrik Hein Lauridsen

**Affiliations:** ^1^ Research Unit for Clinical Biomechanics, Department of Sports Science and Clinical Biomechanics, University of Southern Denmark, Odense, Denmark; ^2^ Department of Orthopaedic Surgery and Traumatology, Odense University Hospital, Odense, Denmark; ^3^ Department of Clinical Research, University of Southern Denmark, Odense, Denmark; ^4^ Chiropractic Knowledge Hub, University of Southern Denmark, Odense, Denmark

**Keywords:** markerless motion capture, reliability, children, kinematics, lower extremity

## Abstract

The clinimetric properties of new technology should be evaluated in relevant populations before its implementation in research or clinical practice. Markerless motion capture is a new digital technology that allows for data collection in young children without some drawbacks commonly encountered with traditional systems. However, important properties, such as test-retest reliability, of this new technology have so far not been investigated. We recorded 63 preschool children using markerless motion capture (The Captury GmbH, Saarbrüken, Germany) while they performed squats and standing broad jumps. A retest session was conducted after 1 week. Recordings from the test session were processed twice to estimate the software-driven instrumental variability. Recordings from the first and second test sessions were compared to evaluate the week-to-week test-retest reliability. Statistical tests included 95% limits of agreement and intraclass correlations of absolute agreement (ICC). Jump length performance and four kinematic variables demonstrated acceptable instrumental variability (ICC > 0.76). The week-to-week reliability was excellent for jump length performance (ICC = 0.90) but poor to moderate (ICC < 0.55) for the kinematic variables. Our results indicate that preschool children exhibit considerable intra-individual kinematic variation from week-to-week during jump landings and squats. Consequently, we suggest that future work should explore individuals with persistent extreme kinematics over multiple test-sessions.

## Introduction

Lower extremity musculoskeletal pain may start early in life (1) and can impact later-in-life health, health behaviors, and career choices (2). Therefore, early detection of children at risk of musculoskeletal pain is of primary concern to reduce the overwhelming worldwide burden of musculoskeletal disorders (3).

Some lower-extremity frontal plane movement patterns in adults and adolescents may be associated with an increased risk of injury or the development of certain pain syndromes (4–6). However, it is unknown if similar movement patterns play a role in developing lower extremity musculoskeletal pain early in life. Investigating a potential association between early-in-life movement patterns and future injury risk requires objective and reliable kinematic measures and an establishment of normative data in relevant age bands. Until now, the practical feasibility of obtaining the necessary kinematic measures has been low, as this has required the use of costly laboratory-based 3D optoelectronic marker-based systems with long participant preparation times. Furthermore, the value of kinematic measures from these systems is likely reduced in young children, as the practice of attaching markers introduces the risk of interfering with the natural movement patterns of the investigated subject (7). However, it is now possible to obtain kinematic measures using portable markerless motion capture systems that require little or no participant preparation time and reduces interference with natural movement (7).

The choice of appropriate technical equipment and algorithms for accurate markerless motion capture is critical (8). One such system measuring preschool children has recently shown valid results for selected kinematics and gross motor performance measures (9). Therefore, quantifying early-in-life movement patterns and gross motor performance may now be feasible using markerless motion capture technology.

Besides validity, the clinimetric properties of the new technology, such as test-retest reliability, should also be evaluated in preschool children before its implementation in research or clinical practice. The main components of error in assessing test-retest reliability are (a) systematic bias and (b) random error due to biological and instrumental variability (10, 11). Markerless systems increasingly utilize deep-learning algorithms to identify the skeletal structure and posture of the investigated subjects (7, 12). If these algorithms contain stochastic processes, any measurement affected by the algorithms will add some random error and thereby increase the overall instrumental variability of the markerless system. The magnitude of this random error is of particular interest when the systems are used to measure preschool children since the algorithms are typically trained using datasets that mostly contain adults, e.g., the widely used MPII dataset (13). Therefore, exploring the software-driven random error associated with markerless measurements in young children is relevant before investigating the clinimetric properties and the clinical relevance of these measurements.

Hence, the research aims of this study were (a) to estimate and evaluate the software-driven instrumental variability associated with objective measures of gross motor function, obtained using a novel markerless motion capture system in preschool children performing squats and jumps, and (b) to estimate the week-to-week test-retest reliability of any variable found to have acceptable software-driven instrumental variability.

## Methods

### Participants and facilities

The research program Motor skills in Pre-school (MiPS) follow a cohort of 865 children with yearly test sessions (14). One of the aims of this cohort is to establish population-based reference data on motor skills in 3–6-year-olds. Seventy-seven children (38 male; 39 female), already included in the MiPS study and attending four different kindergartens, were invited to participate in a retest session scheduled 1 week after their yearly test. We aimed to include at least 50 subjects with complete data as recommended as a minimum sample size to obtain acceptable confidence intervals around estimated reliability parameters (15).

The project was approved by The Regional Committee on Health Research Ethics for Southern Denmark (project ID: S-20150178).

### Physical tests

The present study reports specifically on kinematic and spatial measurements captured using markerless motion capture technology while the children performed squats and standing broad jumps as a part of the overall MiPS testing protocol (14). Both the standing broad jump and the squat test are frequently used in test batteries to assess movement quality with the aim of preventing injury (16). We chose the standing broad jump test as it is a simple, functional test that captures physical performance naturally occurring in human locomotion (17). Furthermore, landings from jumps with a horizontal component produce higher strain on tendons (18) and more extreme kinematics than landings from other types of jumps (18, 19). Such kinematic measurements may have value in future investigations into the potential association between movement patterns and musculoskeletal health (18–20). The squat test was chosen because the movements in the descending phase are similar to those in the landing phase of the standing broad jump and because children, regardless of their level of motor skill, would be able to perform the test.

In both test sessions, the same clinician instructed the children to perform two deep squats and two standing broad jumps using pre-rehearsed and standardized instructions. The children performed the movements barefooted on a 2 cm thick foam mat and practiced all activities at least once before each test session. The children performed the standing broad jumps by placing their feet on two stars marked on the foam mat and jumping as far forward as possible with both feet and without touching the mat with their hands. The children were not required to stand still after landing. The children performed the squats by following visual and oral instructions given by the instructor, who squatted to approximately 110 degrees of knee flexion with the arms stretched out in front of the body. There were no restrictions on foot positions, foot direction, or stance width as the children initiated the squat.

### Equipment, calibration, and post-processing

In both test sessions, the children were recorded using a CapturyLive (The Captury GmbH, Saarbrüken, Germany) (21) markerless motion capture system. This passive vision system (7) scales a subject-specific 3D body model by fitting a template skeleton onto multiple image silhouettes generated *via* a background subtraction procedure (22). The human body is modeled *via* estimates of joint center positions using local optimization procedures and a “sums of spatial Gaussians” approach (22). A best-fit pose for each frame is obtained using generative fitting (7) and forms the basis of the subsequent tracking. Both the scaling and tracking features are based on a proprietary method that uses deep-learning (12) techniques to identify and describe the posture of a human subject within a given frame, similar to the OpenPose library (23). The template skeleton contains, among others, joint-centers of big toes, ankles, knees, and hips. The positions of these joint-centers can be exported using standard export procedures in the software (24).

The validity of the Captury system has been examined against a Vicon optoelectrical motion capturing system using the standard Plug-in-Gait marker model in our laboratory in a convenience sample of 14 preschool children performing standing broad jumps and squats (9). The markerless system was found to measure jump length, knee-to-hip separation ratio (25) (KHR), ankle-to-hip separation ratio (25) (AHR), and knee-to-ankle separation ratio (26) (KASR) with acceptable concurrent validity, while measurements of knee-flexion should be interpreted with care (9, 27). For knee-flexion, the between system reliability and precision estimates ranged from “Excellent” to “Moderate”, and “Good” to “Invalid”, respectively. This span reflects that the precision of the knee flexion measurements dropped to unacceptable levels when the knees were nearly extended (i.e., upright standing) (9).

The current setup of the markerless motion capture system consisted of eight tripod-mounted Blackfly 808 × 608 50 fps digital cameras connected *via* ethernet to a consumer-grade PC with an NVIDIA GeForce GTX 1070 GPU. The calibration followed the documentation manual and was only accepted if the reprojection error of the calibration target was below 2 mm. The eight cameras were placed following a standardized setup ensuring optimal viewpoints from multiple angles.

Post-processing of the recordings in the form of rescaling and retracking (24) was done using CapturyLive version 1.0.163 (21), with the settings “very high” and “multipass” for the squats and “very high” and “simple backward pass” for the standing broad jumps.

### Included variables

**Figure 1:**
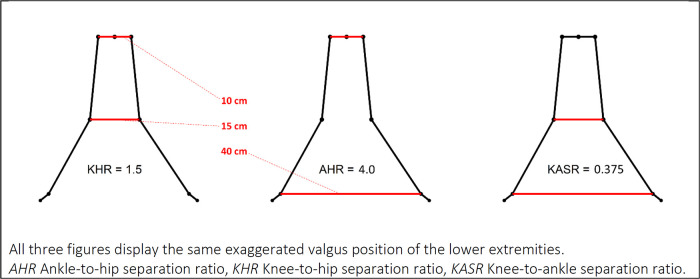
Frontal plane kinematic variables. All three figures display the same exaggerated valgus position of the lower extremities. AHR, ankle-to-hip separation ratio; KHR, knee-to-hip separation ratio; KASR, knee-to-ankle separation ratio.

We exported measures of knee flexion and 3D joint-center positions from the Captury system and calculated jump length and three frontal plane variables (Figure 1) using custom code available on GitHub in the “mocapr” R-package (28). The frontal plane was defined as the plane between the two hip-joint centers perpendicular to the ground plane.

Knee-to-Hip separation Ratio (KHR) (25, 29) is calculated in the frontal plane as the distance between the knee joint centers divided by the distance between the hip joint centers. KHR has also been referred to as the normalized knee separation distance (25, 29) and the normalized knee separation ratio (6).

Ankle-to-Hip separation Ratio (AHR) (25, 29) is calculated in the frontal plane as the distance between the ankle joint centers divided by the distance between the hip joint centers.

Knee-to-Ankle Separation Ratio (KASR) (26) is calculated in the frontal plane as the distance between the knee joint centers divided by the distance between the ankle joint centers. KASR has been suggested as an improved approach to KHR and AHR (26) and has demonstrated a strong relationship with 3D measures of knee abduction angle when measured at the initial contact of a drop-vertical-jump landing (30).

Jump length was calculated using the 3D global coordinate positions of the ankle joints at take-off and impact.

Sample data and custom R code to calculate frontal plane kinematics, jump length, and extracting point-values is available in the R-package “mocapr” (28), downloadable from GitHub.

### Data reduction

We reduced the data for both movements to only reflect the functional points of interest. The squats were reduced to only contain the descending phase and the deepest position, and the jumps were reduced to only include the impact, the descending phase, and the deepest position of the landings.

For each variable, maximum and minimum peak values were extracted. Additionally, two event-specific point values were also extracted from each of the movements. For the squats, the two selected events were the frame where the subject was in the mid-descent position (i.e., halfway down) and the frame in which the subject was in the deepest position. For the jumps, the two selected events were the frame marked with impact and the frame where the subject was in the deepest position of the landing.

A priori, we defined the following criteria for selecting trials for subsequent analysis: The longest jump from each test session would be used, and the squat trial from each test session with a peak knee flexion closest to 110 degrees (according to the instructions) would be used. All analyses of unilateral kinematic variables used the left lower extremity.

### Instrumental variability and week-to-week test-retest reliability

To assess the software-driven instrumental variability, we exported the processed data from the first test session. Subsequently, all scaling and tracking from the first test session were removed, and the raw recordings were processed again with identical settings. We then estimated the software-driven instrumental variability by comparing measurements from the two exports.

The week-to-week test-retest reliability was estimated by comparing the original exports from the first test session with exports from the retest session.

### Data analysis

Data were analyzed using R version 3.6.1 (31), and the “tidyverse” (32) and “psych” (33) R-packages. To avoid misleading reliability coefficients driven by outliers, a conservative approach to describe outliers as more than three standard deviations (SD) away from the mean was chosen (34), and outliers were subsequently removed from further analysis. All compared values were explored for heteroscedasticity using Bland-Altman plots, and the assumption of normality of the differences was examined using QQ-plots. The analysis of reliability was done using intraclass correlation coefficients of absolute agreement [ICC(2,1)A] that we interpreted as being poor (<0.5), moderate (<0.75), good (<0.9), or excellent (>0.9), as suggested by Koo and Li (35). The analysis of agreement was carried out using 95% limits of agreement (LOA) calculated as the mean difference ±1.96 x SD of the difference (36), and the smallest detectable change at the 95% confidence level (SDC) calculated as the SD of the difference x 1.96 (37).

The instrumental variability of the kinematic variables was considered as acceptable if the ICC estimates were good or excellent and the LOAs were between −10° and +10° for knee flexion, between −0.25 and 0.25 for the variables KASR, KHR, AHR, and between −5 and +5 cm for jump length. These limits were based on subjective clinical judgment and knowledge of joint range of motion.

This study follows the GRASS guidelines for reporting reliability and agreement studies (38).

## Results

**Figure 2:**
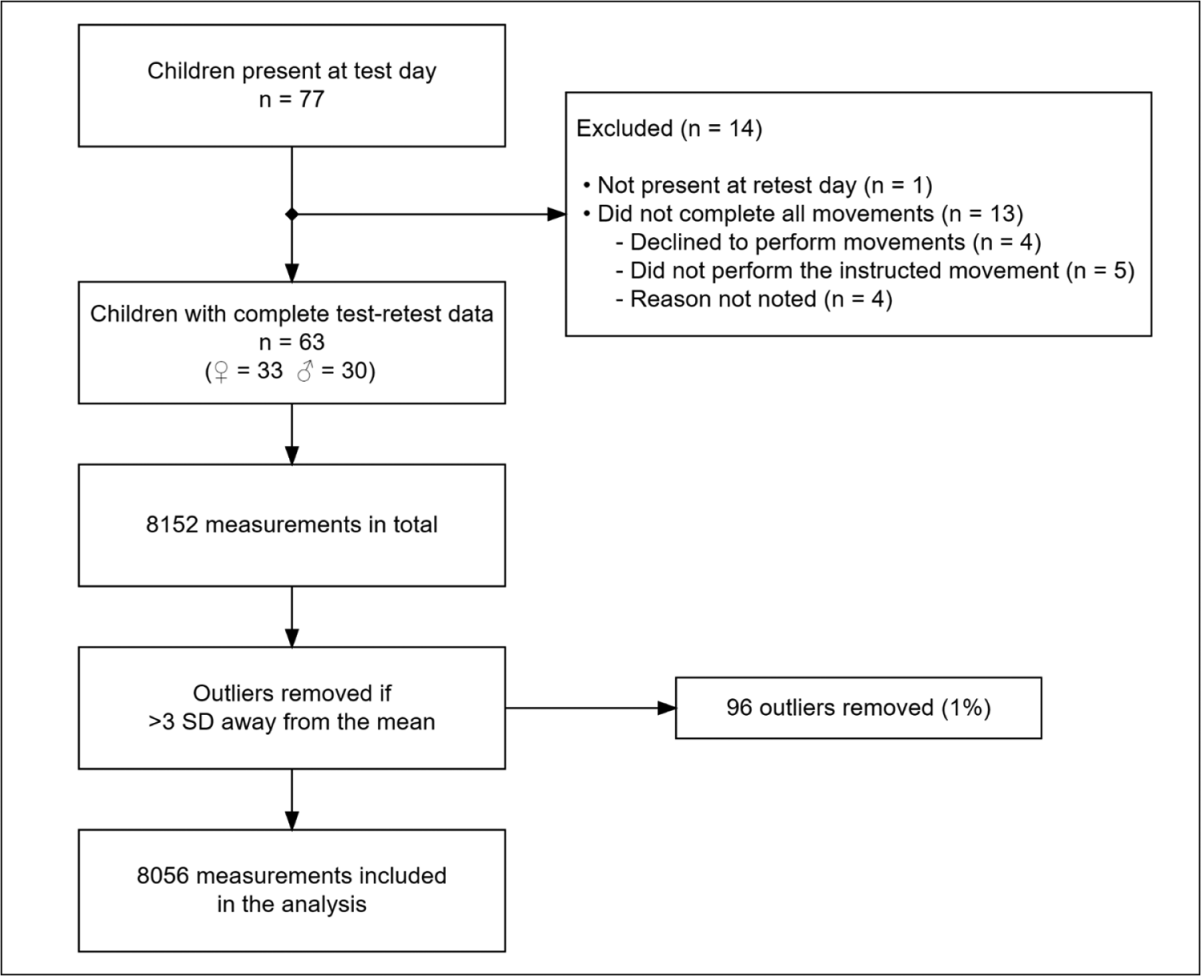
Flowchart of eligible subjects and measurements.

Of the 77 invited children, one child was not present on the retest day, while 13 children were present at both test days but did not have two squats and two jumps recorded on both test days, leaving 33 girls and 30 boys for analyses (Figure 2). The children had a mean age of 5.15 years (SD: 0.79), a mean weight of 19.9 kg (SD: 3.42), and a mean height of 111.23 cm (SD: 11.69).

Of the 8,152 measurements, 96 were identified as outliers and omitted from further analysis. Generally, the included variables were homoscedastic, and the error distribution was normal. Consequently, no variables were transformed.

**Figure 3:**
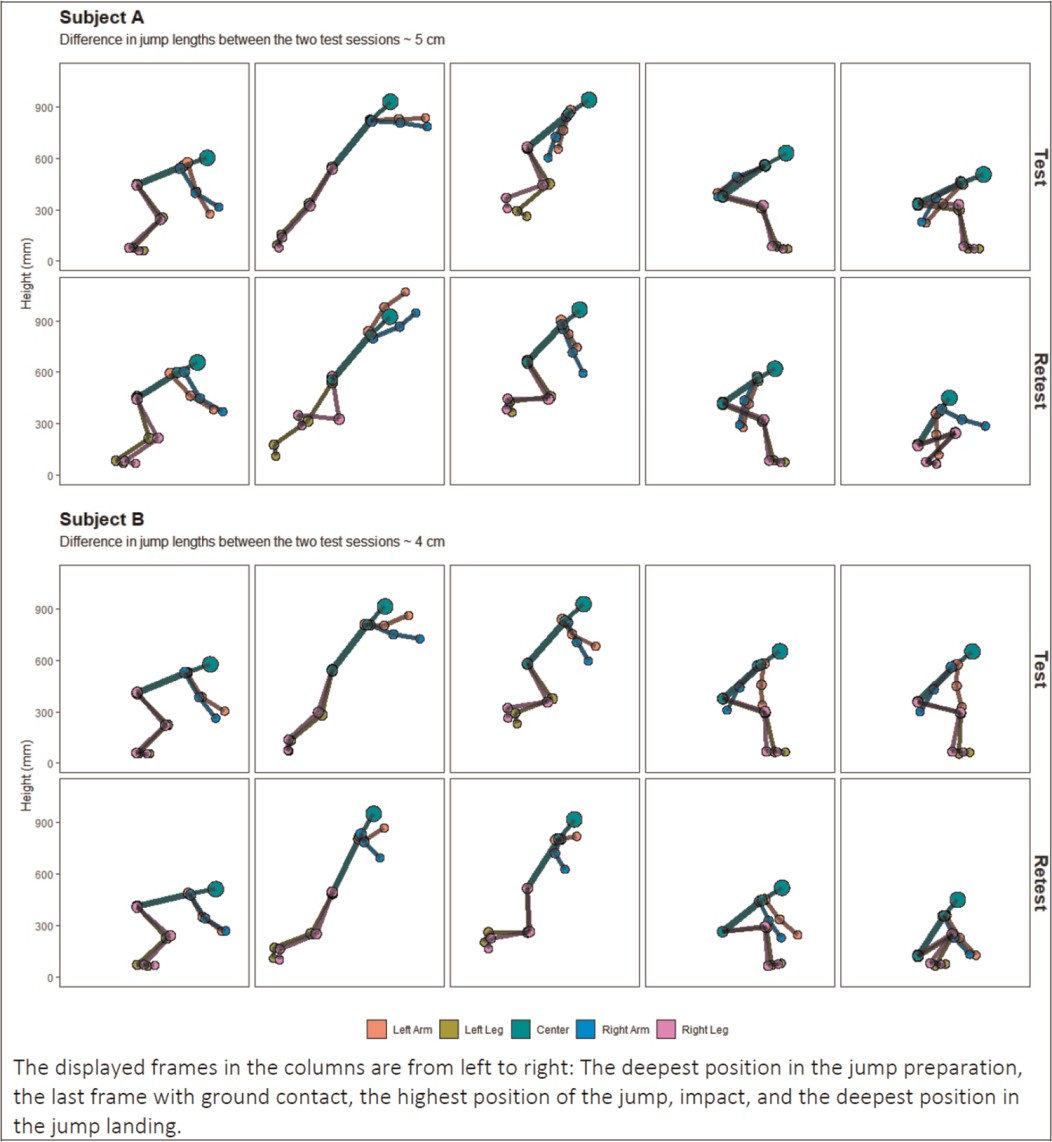
Two representative subjects (**A** and **B**) demonstrating sizeable week-to-week kinematic landing variability between the two test sessions, despite similar jump lengths.

**Figure 4:**
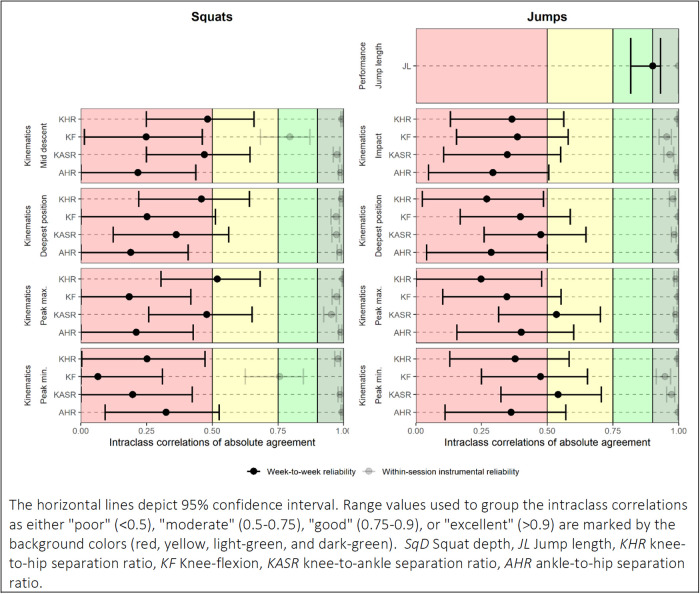
Week-to-week test-retest reliability of performance and kinematic variables captured in 63 preschool children performing squats and jumps. The horizontal lines depict 95% confidence interval. Range values used to group the intraclass correlations as either “poor” (<0.5), “moderate” (0.5–0.75), “good” (0.75–0.9), or “excellent” (>0.9) are marked by the background colors (red, yellow, light-green, and dark-green). SqD, squat depth; JL, jump length; KHR, knee-to-hip separation ratio; KF, knee-flexion; KASR, knee-to-ankle separation ratio; AHR, ankle-to-hip separation ratio.

In the examination of the software-driven instrumental variability, the estimated ICC(2,1)A for jump performance was excellent, the mean difference was 0.17 cm, and the LOA were between −1.69 and 2.04 cm (see Supplementary Table S1). In general, all ICC(2.1)A estimates of software-driven instrumental variability for the kinematic variables were excellent. However, ICC(2.1)A estimates of knee flexion, measured during the start of the squat movements (peak min. and mid-descent), only reached good levels (0.76 and 0.79). The estimated LOA's for all four kinematic variables were found to be acceptable according to our predefined criteria (see Supplementary Table S1). The week-to-week test-retest ICC(2.1)A coefficient for jump length was excellent (0.9). The mean difference was 0.86 cm, while the LOA were somewhat wide (−15.36 to 17.07 cm) (Table 1). We found large week-to-week variations in both the squat and the jump-landing kinematics. Test-retest differences in gross-motor patterns are exemplified in Figure 3, showing two subjects with similar jump length performance (≤5 cm) but markedly different gross-motor landing strategies between the two test sessions. ICC(2.1)A coefficients of week-to-week test-retest reliability were mostly poor (<0.5), with some estimates of KHR and KASR being moderate (between 0.52 and 0.54) (Table 1 and Figure 4). The difference between the ICC(2.1)A estimates of the software-driven instrumental variability and the week-to-week test-retest reliability are visible in Figure 4. The estimated SDC's were large, and the spans of the LOA's were wide for all kinematic variables examined in the analysis of test-retest reliability (Table 1).

**Table 1:** Week-to-week test-retest reliability and agreement of 4 kinematic variables and jump length measured in 63 preschool children using markerless motion capture equipment.

Variable (unit)	Measure		Session mean (SD)		Between session reliability and agreement				
			First	Second	ICC (2.1)A [95% CI]	MD	LLOA	ULOA	SDC
AHR (ratio)	Jump	Deepest position	1.39 (0.47)	1.33 (0.39)	0.17 [−0.08 to 0.40]	0.05	−1.04	1.15	1.10
		Impact	1.41 (0.46)	1.31 (0.36)	0.23 [−0.01 to 0.46]	0.10	−0.90	1.09	1.00
		Peak max.	1.42 (0.48)	1.34 (0.42)	0.20 [−0.07 to 0.44]	0.07	−1.05	1.19	1.12
		Peak min.	1.37 (0.49)	1.31 (0.40)	0.15 [−0.12 to 0.40]	0.06	−1.09	1.21	1.15
	Squat	Deepest position	1.19 (0.38)	1.02 (0.38)	0.19 [−0.04 to 0.41]	0.17	−0.77	1.11	0.94
		Mid-descent	1.06 (0.34)	0.94 (0.35)	0.22 [−0.02 to 0.44]	0.12	−0.73	0.97	0.85
		Peak max.	1.31 (0.33)	1.13 (0.34)	0.19 [−0.04 to 0.41]	0.18	−0.65	1.00	0.83
		Peak min.	0.92 (0.32)	0.80 (0.37)	0.33 [0.10 to 0.54]	0.12	−0.64	0.89	0.77
KASR (ratio)	Jump	Deepest position	1.01 (0.34)	0.96 (0.26)	0.47 [0.25 to 0.64]	0.05	−0.57	0.67	0.62
		Impact	0.90 (0.19)	0.90 (0.18)	0.39 [0.16 to 0.59]	0.00	−0.40	0.40	0.40
		Peak max.	1.03 (0.31)	0.99 (0.25)	0.54 [0.32 to 0.70]	0.04	−0.49	0.58	0.53
		Peak min.	0.92 (0.22)	0.91 (0.20)	0.55 [0.33 to 0.71]	0.00	−0.39	0.40	0.40
	Squat	Deepest position	1.55 (0.58)	1.54 (0.61)	0.36 [0.12 to 0.56]	0.01	−1.32	1.34	1.33
		Mid-descent	1.32 (0.45)	1.18 (0.32)	0.34 [0.10 to 0.54]	0.14	−0.74	1.02	0.88
		Peak max.	1.87 (0.71)	1.92 (0.77)	0.48 [0.26 to 0.65]	−0.05	−1.54	1.44	1.49
		Peak min.	0.87 (0.19)	0.85 (0.16)	0.20 [−0.05 to 0.42]	0.01	−0.41	0.44	0.43
KHR (ratio)	Jump	Deepest position	1.35 (0.38)	1.25 (0.42)	0.22 [−0.03 to 0.44]	0.09	−0.89	1.08	0.98
		Impact	1.25 (0.31)	1.19 (0.29)	0.26 [0.01 to 0.47]	0.07	−0.65	0.78	0.71
		Peak max.	1.37 (0.37)	1.28 (0.41)	0.22 [−0.04 to 0.45]	0.10	−0.86	1.05	0.96
		Peak min.	1.23 (0.33)	1.19 (0.32)	0.32 [0.06 to 0.54]	0.04	−0.70	0.78	0.74
	Squat	Deepest position	1.78 (0.59)	1.54 (0.60)	0.52 [0.29 to 0.69]	0.24	−0.85	1.33	1.09
		Mid-descent	1.36 (0.56)	1.09 (0.46)	0.47 [0.20 to 0.66]	0.27	−0.71	1.24	0.98
		Peak max.	1.99 (0.76)	1.75 (0.62)	0.52 [0.30 to 0.68]	0.24	−1.06	1.53	1.29
		Peak min.	0.91 (0.14)	0.78 (0.21)	0.27 [0.02 to 0.49]	0.12	−0.27	0.52	0.40
Knee flexion (°)	Jump	Deepest position	85.65 (28.54)	81.65 (18.01)	0.43 [0.20 to 0.61]	4.01	−46.07	54.08	50.07
		Impact	53.41 (11.79)	53.21 (10.69)	0.33 [0.08 to 0.53]	0.20	−25.54	25.93	25.73
		Peak max.	85.15 (27.01)	81.10 (19.58)	0.34 [0.09 to 0.55]	4.06	−48.93	57.04	52.98
		Peak min.	66.81 (12.57)	65.59 (10.09)	0.43 [0.20 to 0.62]	1.23	−22.62	25.08	23.85
	Squat	Deepest position	154.70 (10.17)	144.72 (7.95)	0.25 [−0.06 to 0.51]	9.98	−9.63	29.58	19.61
		Mid-descent	99.67 (6.45)	94.66 (9.18)	0.25 [0.01 to 0.46]	5.02	−13.44	23.47	18.46
		Peak max.	155.01 (9.61)	144.78 (8.23)	0.18 [−0.06 to 0.42]	10.24	−10.47	30.95	20.71
		Peak min.	5.40 (3.89)	4.51 (3.66)	0.11 [−0.14 to 0.35]	0.90	−8.96	10.75	9.86
Jump length (cm)			92.88 (20.27)	92.02 (17.47)	0.90 [0.85 to 0.94]	0.86	−15.36	17.07	16.22

SD, standard deviation; ICC(2.1)A, intraclass correlation of absolute agreement; 95% CI, 95% confidence interval; MD, mean difference; LLOA, lower limit of agreement; ULOA, upper limit of agreement; SDC, smallest detectable change; AHR, ankle-to-hip separation ratio; KASR, knee-to-ankle separation ratio; KHR, knee-to-hip separation ratio.

## Discussion

This study utilized novel markerless motion capture technology and is, to our knowledge, the first to report on the week-to-week test-retest reliability of jump-landing and squat kinematics captured in young children. The software-driven instrumental variability of our data-collection method was acceptable for the measures of jump length and for the kinematic variables. The measures of jump length were similar between the two test sessions with narrow LOA's, negligible mean differences, and good to excellent ICC(2.1)A estimates. In contrast, the test-retest reliability of all examined kinematic variables was low, indicating that high levels of intra-individual variation in the landing and squatting kinematics are normal in preschool children.

The kinematic variables examined in this study were found to have low levels of within-session software-driven instrumental variability, although the software utilizes stochastic processes. Indicating that the observed large kinematic differences between the two test sessions reflect actual/biological differences in the kinematic motor patterns of the children.

The week-to-week test-retest reliability of standing broad jump performance has previously been examined by Román et al. in a sample of 90 3-6-year-old children (39). Similar to this study, they estimated the ICC to be 0.913 (ICC type not specified). However, the span of their reported LOA was from −21.4 to 25.4 cm (39), which is 14.3 cm or 44% wider than ours. Since our results are comparable to previous research, we believe that the children in our study were exhibiting normal levels of intraindividual between-session variability in their jump length performance.

All kinematic variables were found to have poor or low-end moderate week-to-week test-retest ICC(2,1)A estimates, the SDC's were large, and the span of the LOA's wide. When determining the between-session test-retest reliability, the two sources of variability, instrumental variability, and biological variability, are unavoidably combined (11). This fact calls for careful consideration in the interpretation of our results. We tested the week-to-week test-retest reliability of five variables. The five variables have demonstrated acceptable validity in our previous work (9) and acceptable within-session software-driven instrumental variability in the current study. Of the five variables, measures of jump length were found to have excellent week-to-week test-retest reliability. However, the week-to-week test-retest reliability of the kinematic variables was poor. Together, these results indicate that the poor week-to-week test-retest scores for the kinematic variables reflect high intra-individual biological variation in the movement patterns of squats and jump-landings in preschool children. Our results therefore indicate that typically developing children exhibit considerable variation in their kinematic movement patterns when jumping and squatting. Consequently, we find that *single* kinematic measures obtained from these movements are unlikely to hold clinical value.

It is unknown to what extent pathology can alter the movement variability of preschool children, but several studies have noted that pathology can reduce the magnitudes of movement variability in a range of other populations (40–43). Therefore, we suggest that future work into the possible relationship between early-in-life motor patterns and later-in-life musculoskeletal health should explore individuals with persistent extreme kinematics over repeated test sessions.

## Limitations

This study examined the natural movement patterns of the children, and thus we only gave minimal instructions to the children on how to perform the movements. Stricter criteria and more elaborate instructions may improve the between-session test-retest reliability, but could also alter the natural movement patterns of the children.

We assessed the software-driven instrumental variability of the motion capture equipment by re-analyzing the same recordings twice. This approach allowed us to assess the variability associated with the software, which we believe is the most error-prone part of the CapturyLive system and for markerless motion capture systems in general. Nevertheless, other sources of instrumental variation could also have affected our data, e.g., the illumination of the recording area was difficult to standardize as some of the gym halls had windows that could not be screened for sunlight.

We used 8 digital cameras in the markerless motion capture setup for the current study. This number of cameras was recommended in early research that found the use of setups with less than 8 cameras for accurately capturing human movement to be questionable (44). Given the rapid development of markerless motion capture, it may be that simpler and more feasible systems with fewer cameras have the same acceptable levels of software-driven instrumental variability today as the 8 camera setup we evaluated in this experiment. This, however, should be evaluated independently in future studies.

The children in the present sample were enrolled in the MiPS study. In the MiPS study, all children attending public preschools in the Municipality of Svendborg, Denmark, were invited to participate (14). Consequently, we expect most of these children to be typically developing healthy children. However, our sample may include children with pathologies if these pathologies would not prevent the children from attending a public preschool in Denmark.

## Conclusion

Estimates of jump length, KHR, AHR, KASR, and knee flexion can be measured in young children with acceptable software-driven instrumental variability using commercially available markerless motion capture technology. Jump length can be measured using the same system with excellent week-to-week test-retest reliability.

The children in the present representative sample of preschool children exhibited considerable intra-individual biological variation in their kinematic movement patterns when they landed from jumps and performed squats. Therefore, any *single* kinematic measure from these movements in this population is unlikely to be of clinical value. Consequently, we suggest that future work into the possible relationship between early-in-life motor patterns and later-in-life musculoskeletal health should explore individuals with persistent extreme kinematics over repeated test-sessions.

## Data Availability

The raw data supporting the conclusions of this article will be made available by the authors, without undue reservation.
