# Supplementary material for: Test-retest reliability and agreement of lower-extremity kinematics captured in squatting and jumping preschool children using markerless motion capture technology
Source: Front Digit Health. 2022 Dec 5;4:1027647. doi: 10.3389/fdgth.2022.1027647 (PMC9760710; doi:10.3389/fdgth.2022.1027647)
Supplement: Supplementary file 1 [file Table1.docx]

| **Supplementary Table** Software-driven **i**nstrumental variability of jump length and four kinematic variables captured in 63 pre-school children using markerless motion capture equipment. | | | | | | | | | |
| --- | --- | --- | --- | --- | --- | --- | --- | --- | --- |
|  | | | | | | | | | |
|  | | | **mean (SD)** | | **Between export**  **Reliability and Agreement** | | | | |
| **Variable (unit)** | **Measure** | | **First Export** | **Second Export** | **ICC(2.1)A [95% CI]** | **MD** | **LLOA** | **ULOA** | **SDC** |
| **Jump length (cm)** |  | | 92.12 (20.92) | 91.95 (20.94) | 1.00 [1.00 to 1.00] | 0.17 | -1.69 | 2.04 | 1.86 |
| **AHR (ratio)** | jump | Deepest position | 1.39 (0.47) | 1.39 (0.48) | 1.00 [ 1.00 to 1.00] | 0.00 | -0.06 | 0.06 | 0.06 |
|  |  | Impact | 1.40 (0.46) | 1.41 (0.45) | 1.00 [ 0.99 to 1.00] | -0.01 | -0.10 | 0.08 | 0.09 |
|  |  | Peak max. | 1.41 (0.47) | 1.41 (0.47) | 1.00 [ 0.99 to 1.00] | 0.00 | -0.09 | 0.08 | 0.09 |
|  |  | Peak min. | 1.36 (0.48) | 1.36 (0.48) | 1.00 [ 1.00 to 1.00] | 0.00 | -0.06 | 0.06 | 0.06 |
|  | squat | Deepest position | 1.19 (0.38) | 1.19 (0.39) | 0.98 [ 0.97 to 0.99] | 0.00 | -0.13 | 0.13 | 0.13 |
|  |  | Mid-descent | 1.07 (0.34) | 1.07 (0.35) | 0.99 [ 0.98 to 0.99] | 0.00 | -0.11 | 0.11 | 0.11 |
|  |  | Peak max. | 1.31 (0.33) | 1.31 (0.34) | 0.99 [ 0.98 to 0.99] | 0.00 | -0.10 | 0.10 | 0.10 |
|  |  | Peak min. | 0.95 (0.34) | 0.95 (0.36) | 0.99 [ 0.99 to 1.00] | 0.00 | -0.08 | 0.08 | 0.08 |
| **KASR (ratio)** | jump | Deepest position | 1.01 (0.34) | 1.00 (0.35) | 0.99 [ 0.98 to 0.99] | 0.01 | -0.10 | 0.11 | 0.11 |
|  |  | Impact | 0.90 (0.19) | 0.89 (0.18) | 0.98 [ 0.96 to 0.99] | 0.01 | -0.07 | 0.09 | 0.08 |
|  |  | Peak max. | 1.03 (0.31) | 1.03 (0.32) | 0.99 [ 0.98 to 0.99] | 0.00 | -0.10 | 0.09 | 0.10 |
|  |  | Peak min. | 0.93 (0.22) | 0.91 (0.22) | 0.97 [ 0.95 to 0.98] | 0.01 | -0.08 | 0.11 | 0.10 |
|  | squat | Deepest position | 1.54 (0.58) | 1.54 (0.59) | 0.97 [ 0.95 to 0.98] | 0.00 | -0.26 | 0.27 | 0.27 |
|  |  | Mid-descent | 1.37 (0.51) | 1.36 (0.50) | 0.98 [ 0.96 to 0.98] | 0.01 | -0.21 | 0.23 | 0.22 |
|  |  | Peak max. | 1.89 (0.74) | 1.91 (0.81) | 0.95 [ 0.92 to 0.97] | -0.02 | -0.48 | 0.45 | 0.47 |
|  |  | Peak min. | 0.87 (0.19) | 0.87 (0.19) | 0.99 [ 0.98 to 0.99] | 0.00 | -0.06 | 0.06 | 0.06 |
| **KHR (ratio)** | jump | Deepest position | 1.35 (0.38) | 1.34 (0.38) | 0.98 [ 0.97 to 0.99] | 0.01 | -0.14 | 0.15 | 0.14 |
|  |  | Impact | 1.27 (0.33) | 1.27 (0.33) | 1.00 [ 0.99 to 1.00] | 0.00 | -0.05 | 0.06 | 0.06 |
|  |  | Peak max. | 1.37 (0.36) | 1.37 (0.36) | 0.99 [ 0.98 to 0.99] | 0.00 | -0.11 | 0.11 | 0.11 |
|  |  | Peak min. | 1.24 (0.33) | 1.23 (0.32) | 1.00 [ 0.99 to 1.00] | 0.01 | -0.05 | 0.07 | 0.06 |
|  | squat | Deepest position | 1.81 (0.62) | 1.79 (0.62) | 0.99 [ 0.98 to 0.99] | 0.02 | -0.14 | 0.18 | 0.16 |
|  |  | Mid-descent | 1.41 (0.61) | 1.40 (0.60) | 0.99 [ 0.99 to 1.00] | 0.01 | -0.13 | 0.15 | 0.14 |
|  |  | Peak max. | 1.99 (0.76) | 1.98 (0.74) | 1.00 [ 0.99 to 1.00] | 0.02 | -0.11 | 0.14 | 0.13 |
|  |  | Peak min. | 0.92 (0.16) | 0.92 (0.15) | 0.98 [ 0.97 to 0.99] | 0.00 | -0.06 | 0.06 | 0.06 |
| **Knee flexion (°)** | jump | Deepest position | 86.27 (28.87) | 86.23 (29.03) | 1.00 [ 1.00 to 1.00] | 0.04 | -3.64 | 3.72 | 3.68 |
|  |  | Impact | 53.50 (11.73) | 53.19 (12.40) | 0.97 [ 0.96 to 0.98] | 0.31 | -5.05 | 5.68 | 5.36 |
|  |  | Peak max. | 84.88 (27.72) | 85.37 (27.67) | 1.00 [ 0.99 to 1.00] | -0.48 | -4.99 | 4.02 | 4.51 |
|  |  | Peak min. | 66.30 (12.28) | 66.27 (12.97) | 0.97 [ 0.96 to 0.98] | 0.04 | -5.58 | 5.65 | 5.62 |
|  | squat | Deepest position | 154.70 (10.17) | 154.07 (9.95) | 0.97 [ 0.95 to 0.98] | 0.62 | -4.00 | 5.25 | 4.63 |
|  |  | Mid-descent | 99.67 (6.45) | 99.15 (6.88) | 0.79 [ 0.68 to 0.87] | 0.52 | -7.85 | 8.89 | 8.37 |
|  |  | Peak max. | 155.01 (9.61) | 154.47 (9.62) | 0.97 [ 0.96 to 0.98] | 0.54 | -3.71 | 4.78 | 4.24 |
|  |  | Peak min. | 5.37 (3.87) | 5.32 (4.63) | 0.76 [ 0.63 to 0.85] | 0.05 | -5.83 | 5.92 | 5.88 |
| **Jump length (cm)** | | | 92.12 (20.92) | 91.95 (20.94) | 1.00 [1.00 to 1.00] | 0.17 | -1.69 | 2.04 | 1.86 |
| **Note:** SD (Standard deviation), ICC(2.1)A (intraclass correlation of absolute agreement); 95% CI (95% confidence interval), MD (mean difference), LLOA (lower limit of agreement), ULOA (upper limit of agreement), SDC (smallest detectable change), AHR (Ankle-to-hip separation ratio), KASR (Knee-to-ankle separation ratio), KHR (Knee-to-hip separation ratio). | | | | | | | | | |
